# Preparation of Sulfobetaine-Grafted PVDF Hollow Fiber Membranes with a Stably Anti-Protein-Fouling Performance

**DOI:** 10.3390/membranes4020181

**Published:** 2014-04-08

**Authors:** Qian Li, Han-Han Lin, Xiao-Lin Wang

**Affiliations:** Membrane Technology & Engineering Research Center, Department of Chemical Engineering, Tsinghua University, Beijing 100084, China; E-Mails: lq517_23@126.com (Q.L.); linhanhan050505@163.com (H.-H.L.)

**Keywords:** two-step polymerization, poly(vinylidene fluoride) membrane, sulfobetaine, anti-protein-fouling performance, stability

## Abstract

Based on a two-step polymerization method, two sulfobetaine-based zwitterionic monomers, including 3-(methacryloylamino) propyl-dimethyl-(3-sulfopropyl) ammonium hydroxide (MPDSAH) and 2-(methacryloyloxyethyl) ethyl-dimethyl-(3-sulfopropyl) ammonium (MEDSA), were successfully grafted from poly(vinylidene fluoride) (PVDF) hollow fiber membrane surfaces in the presence of *N*,*N*′-methylene bisacrylamide (MBAA) as a cross-linking agent. The mechanical properties of the PVDF membrane were improved by the zwitterionic surface layers. The surface hydrophilicity of PVDF membranes was significantly enhanced and the polyMPDSAH-g-PVDF membrane showed a higher hydrophilicity due to the higher grafting amount. Compared to the polyMEDSA-g-PVDF membrane, the polyMPDSAH-g-PVDF membrane showed excellent significantly better anti-protein-fouling performance with a flux recovery ratio (RFR) higher than 90% during the cyclic filtration of a bovine serum albumin (BSA) solution. The polyMPDSAH-g-PVDF membrane showed an obvious electrolyte-responsive behavior and its protein-fouling-resistance performance was improved further during the filtration of the protein solution with 100 mmol/L of NaCl. After cleaned with a membrane cleaning solution for 16 days, the grafted MPDSAH layer on the PVDF membrane could be maintain without any chang; however, the polyMEDSA-g-PVDF membrane lost the grafted MEDSA layer after this treatment. Therefore, the amide group of sulfobetaine, which contributed significantly to the higher hydrophilicity and stability, was shown to be imperative in modifying the PVDF membrane for a stable anti-protein-fouling performance via the two-step polymerization method.

## 1. Introduction

Due to its extraordinary mechanical property, high chemical resistance, and good thermal stability, polyvinylidene fluoride (PVDF) has been recognized as one of the most attractive polymers in the membrane industry and widely used in many separation applications [1,2]. However, PVDF membranes suffer from membrane protein-fouling when used in practical biotechnological applications, such as biological effluent treatment, protein purification, and bacteria filtration, *etc.*, because of its low surface energy and hydrophobic characteristics [3]. Protein-fouling of membranes is considered to be the first step in membrane fouling and is often followed by bacterial infection, thrombus formation, and other undesirable reactions and responses [4,5,6,7,8,9,10]. Therefore, the development of PVDF membranes with a protein adsorption-resistant surface is of great importance [11].

Sulfobetaine, a zwitterionic material, has been proven to be a biocompatible material with excellent protein adsorption-resistance properties [12,13]. Sulfobetaine surfaces are capable of binding a significant amount of water molecules, because of the formation of a hydration layer via electrostatic interactions and hydrogen bonds [14,15]. Many researchers have confirmed that sulfobetaine coatings on various polymer membranes effectively resist protein adsorption and subsequently significantly retard bacterial biofilm formation [3,14,15,16,17,18,19]. Our previous work showed that two typical sulfobetaine monomers, 3-(methacryloylamino) propyl-dimethyl-(3-sulfopropyl) ammonium hydroxide (MPDSAH) and 2-(methacryloyloxyethyl) ethyl-dimethyl-(3-sulfopropyl) ammonium (MEDSA), could be successfully grafted onto the PVDF hollow fiber membrane surface via alkaline treatment and atom transfer radical polymerization (ATRP) [20]. However, alkaline treatment degrades the PVDF membrane and significantly lowers its strength [21]. Thus, a two-step polymerization—a chemically reactive polyHEMA on the surface of the PVDF membrane for the subsequent graft copolymerization with zwitterionic sulfobetaine—has been proposed to maintain the membrane’s strength and increase the grafting amount (GA) [22]. However, possible differences between polyMPDSAH-g-PVDF and polyMEDSA-g-PVDF membranes have not been investigated during a two-step polymerization process. On the other hand, although we previously reported that the electrolyte-responsive behavior of the polyMPDSAH-g-PVDF membrane positively contributes to the anti-fouling performance [23], the electrolyte-responsive effect of polyMEDSA-g-PVDF membrane is still unclear.

In this work, poly(2-hydroxyethyl methacryl ate) (poly(HEMA)) chains were first grafted onto the outer surface of the PVDF membrane via atom transfer radical polymerization (ATRP) to provide the initiation sites for subsequent cerium (Ce (IV))-induced graft copolymerization of sulfobetaine monomers (MPDSAH or MEDSA) in the presence of *N*,*N*′-methylene bisacrylamide (MBAA) as a cross-linking agent. The hydrophilicity, protein-fouling-resistance, electrolyte-responsiveness, and stability of polyMPDSAH-g-PVDF and polyMEDSA-g-PVDF membranes were compared under similar conditions. The surface composition, morphology, and hydrophilicity of the nascent and modified PVDF hollow fiber membranes were characterized by attenuated total reflectance spectrophotometer (ATR-FTIR), X-ray photoelectron spectroscopy (XPS), scanning electron microscope (SEM), and contact angle (CA) goniometer. The anti-fouling properties were determined via cyclic filtration of a protein solution with or without electrolyte. The stability of the modified PVDF membranes was evaluated by examining the surface composition and anti-protein-fouling performance after membrane cleaning.

## 2. Results and Discussion

### 2.1. Surface Composition and Grafting Amount

**Figure 1:**
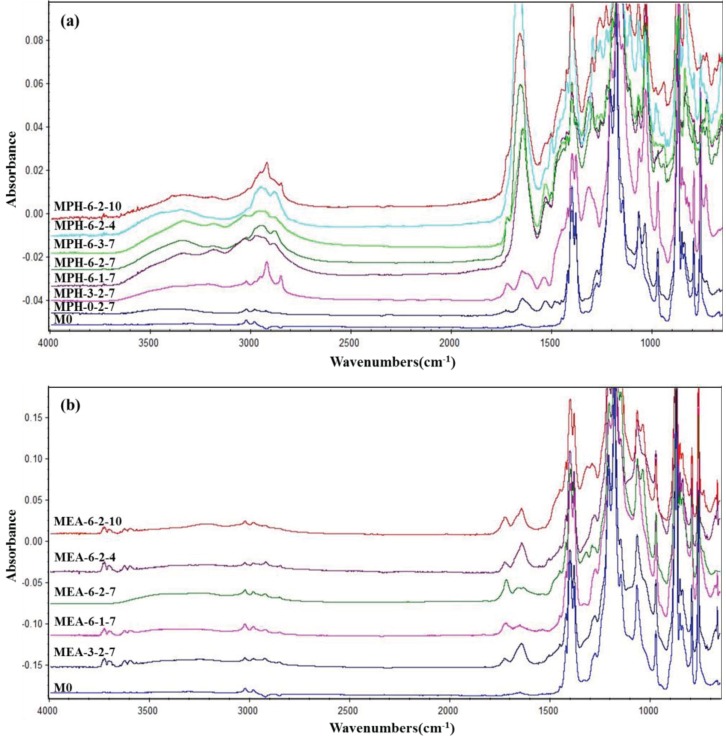
ATR-FTIR spectra of various membranes (**a**) polyMPDSAH-g-PVDF membrane; (**b**) polyMEDSA-g-PVDF membrane.

**Figure 2:**
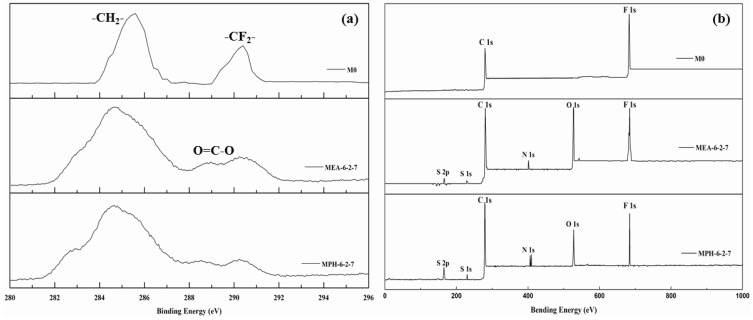
XPS C 1s core-level spectra (**a**) and XPS spectra (**b**) of various membranes.

In this work, two sulfobetaine monomers (MPDSAH and MEDSA) were grafted onto the PVDF hollow fiber membrane surface via a two-step polymerization. The typical spectra of the polyMPDSAH-g-PVDF and polyMEDSA-g-PVDF membranes are displayed in Figure 1 and Figure 2. In Figure 1a, characteristic peaks appeared at 1646 cm^−1^ and 1533 cm^−1^ for the polyMPDSAH-g-PVDF membrane, which are attributed to the bending vibration of amide I (C=O) and amide II (N–H). In Figure 1b, adsorption peaks centered around 1727 cm^−1^ (O–C=O stretching) can be seen from the spectra of the polyMEDSA-g-PVDF membrane. A peak attributed to the stretching vibration of –SO_3_ group, which originates from the side chains of polyMPDSAH and polyMEDSA, appears around 1037 cm^−1^. The distinct peaks that appear around 2850 and 2930 cm^−1^ can be attributed to the symmetric and asymmetric stretching vibrations of the –CH_2_– group originating from the molecular structure of MBAA. Moreover, Figure 1 suggests that the intensities of the characteristic peaks of amide (1646 cm^−1^ and 1533 cm^−1^) and –SO_3_ (1037 cm^−1^) stretching vibration become stronger with the increase of the cross-linking agent and each sulfobetaine monomer. The chemical composition of the membrane surfaces was further determined by XPS analysis, as shown in Figure 2. Figure 2a shows the XPS C 1s core-level spectra processed via curve fitting. The binding energies of –CH_2_– (285.9 eV) and –CF_2_– (290.4 eV) are associated with the chemical structure of PVDF. The peaks with a binding energy of O=C–O (288.8 eV) is associated with the grafted poly(MPDSAH) or poly(MEDSA) chains. From Figure 2b, two major emission peaks at 280.1 and 683.4 eV can be assigned to the binding energies of C 1s and F 1s for the nascent PVDF membrane (M0), respectively. After the grafting polymerization of sulfobetaine monomers onto the outside surface of the PVDF hollow fiber membrane, the characteristic F 1s peak disappears, but the peaks around 230.9 eV (S 1s), 167.4 eV (S 2p), 402.1 eV (N 1s), and 527.5 eV (O 1s) appear on the MPH-6-2-7 and MEA-6-2-7 membrane surface. These results demonstrate both sulfobetaine layers have been successfully fabricated on the outside surface of the PVDF hollow fiber membrane. Additionally, the characteristic peaks of the polyMPDSAH-g-PVDF membrane are stronger than the polyMEDSA-g-PVDF membrane, indicating that the grafting efficiency of the former is higher.

The grafting amount of polyMPDSAH and polyMEDSA can be adjusted by controlling the concentrations of cross-linking agent, sulfobetaine monomer, and initiator. The grafting amount was measured by the weight increase per area after surface modification, which allowed evaluation of the total amount of grafted copolymer on the PVDF hollow fiber membrane surface [20,24]. Table 1 shows the positive correlation effect of the cross-linking agent MBAA concentration on the grafting amount for poly(sulfobetaine)-g-PVDF hollow fiber membranes. The results further prove that MBAA can stimulate both polyMPDSAH and polyMEDSA grafting from the outside surface of PVDF hollow fiber membrane. It also shows that the grafting amount increases with the increase in the sulfobetaine and initiator concentrations. The grafting amount levels off with a further increase at 0.2 mol/L sulfobetaine monomer concentration. The reasons for these observations were discussed in more detail in our previous studies [22,23]. When the cross-linking agent (0.06 mol/L), zwitterionic monomers (MPDSAH or MEDSA; 0.2 mol/L, and initiator (0.07 mol/L) were added during the two-step polymerization, the grafting amount values of MPH-6-2-7 and MEA-6-2-7 were 673.2 μg/cm^2^ and 225.7 μg/cm^2^, respectively. These results are consistent with the discussion on the surface composition of poly(sulfobetaine)-grafted PVDF hollow fiber membranes. The higher molecular weight and the longer molecular chains of MPDSAH contribute to the greater weight increase after grafting polymerization, which can be found in Table 2.

**Table 1:** Grafting amount, mean pore size, and membrane strength of various membranes.

Membrane code	Grafting amount	Mean pore size of membrane surface	Membrane strength		Protein adsorption
			Tensile strength	Elongation ratio	
	µg/cm^2^	µm	Pa	%	µg/cm^2^
M0	–	0.109	3.8	48.4	21.4
MPH-0-2-7	153.2	0.107	3.9	65.8	15.3
MPH-3-2-7	244.7	0.074	4.5	116.4	2.0
MPH-6-1-7	618.7	0.059	4.7	124.8	0
MPH-6-2-7	673.2	0.048	4.7	126.4	0
MPH-6-3-7	680.5	0.048	4.8	126.8	0
MPH-6-2-4	304.1	0.067	3.9	103.9	3.2
MPH-6-2-10	688.0	0.049	4.8	127.9	0
MEA-0-2-7	101.3	0.107	3.7	35.2	15.0
MEA-3-2-7	186.9	0.108	4.9	37.5	15.6
MEA-6-1-7	172.5	0.108	4.0	46.1	15.5
MEA-6-2-7	225.7	0.089	4.4	48.1	5.1
MEA-6-3-7	227.1	0.088	4.6	48.6	5.0
MEA-6-2-4	205.2	0.090	4.3	47.0	6.5
MEA-6-2-10	240.7	0.087	4.8	48.0	4.2

**Table 2:** Properties of MPDSAH and MEDSA.

Sulfobetaine monomer	Molecular structure	Molecular weight	Melting point (°C)	ε_r_ (20–30 °C)
MPDSAH	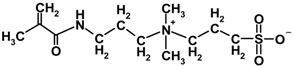	292.39	190	28.18
MEDSA	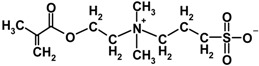	279.35	150~155	7.03

### 2.2. Morphological Mechanical Properties

**Figure 3:**
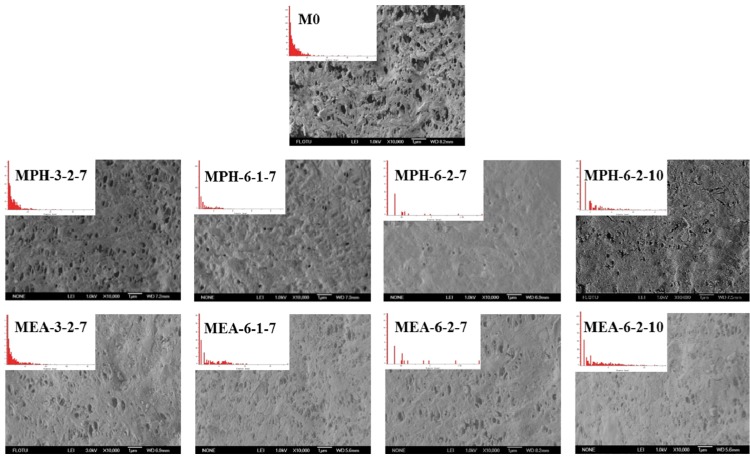
SEM images and pore size distribution of various membranes.

The surface morphologies of various membranes were examined via SEM, as shown in Figure 3. Compared with the nascent PVDF membrane (M0), the opening of the majority of pores at the modified membrane surfaces appeared to be significantly reduced, because the zwitterionic polymer chains covered these after grafting polymerization. It can be seen that MPH-3-2-7, MPH-6-1-7, MEA-3-2-7 and MEA-6-1-7 are only partially covered with grafted zwitterionic polymers. As the GA increases, the pores on the surface of MPH-6-2-7 and MEA-6-2-7 appeared to be fully covered, especially for the former. The surface pore diameter could be characterized by SEM combined with image analysis software. The mean pore size and pore size distribution of various membrane samples are shown in Table 1 and Figure 3. It can be seen that the higher GA corresponds with fewer surface pores. With increasing grafting, the surface pore size distribution of poly(sulfobetaine)-g-PVDF membranes becomes uniform and the mean pore size becomes much smaller. Comparison of the pore size and surface morphology between polyMPDSAH-g-PVDF and polyMEDSA-g-PVDF membranes shows that the former exhibits a denser surface due to the higher grafting amount.

As two important mechanical properties of porous membranes, tensile strength and the elongation ratio were measured, which are shown in Table 1. With the increase in the concentration of cross-linking agent, sulfobetaine monomer, and initiator during the two-step polymerization, the tensile strength and elongation ratio values of sulfobetaine-modified PVDF membranes increase. A dense packing layer of sulfobetaine polymer on the PVDF membrane surface enhances the membrane strength. It is also obvious that the polyMPDSAH-g-PVDF membrane shows much better mechanical properties than polyMEDSA-g-PVDF membranes. The elongation ratio value of the former is twice that of the latter, indicating an increased membrane elasticity. All the results can be attributed to a higher grafting amount and denser membrane surface.

### 2.3. Thermal and Hydrophilic Characterization

**Figure 4:**
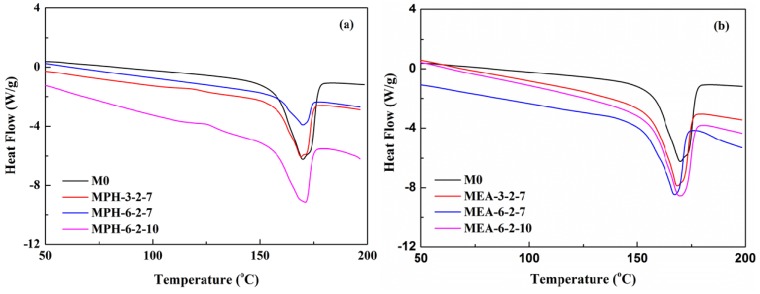
DSC curves of various membranes (**a**) polyMPDSAH-g-PVDF membrane; (**b**) polyMEDSA-g-PVDF membrane.

The thermal properties of various membranes are shown in Figure 4. The Differential scanning calorimetry (DSC) curves in Figure 4a show that the melting temperature increases after grafting polymerization of MPDSAH on the PVDF membrane due to the higher melting point of MPDSAH (190 °C) compared with PVDF (170 °C) [1,20]. This means that the thermal properties of polyMPDSAH-g-PVDF membranes are improved slightly. On the contrary, the thermal properties of polyMEDSA-g-PVDF membranes are reduced due to the lower melting point of MEDSA (150–155 °C) as shown in Table 2. Besides, the difference in the grafting amount of two sulfobetaine-grafted PVDF membranes also contributed to the imporved thermal properties for polyMPDSAH-g-PVDF membranes and the poorer thermal properties for polyMEDSA-g-PVDF membranes.

**Figure 5:**
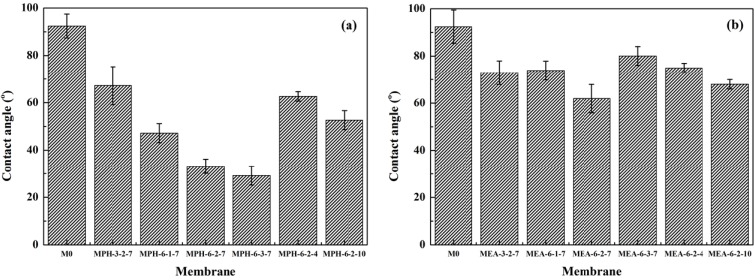
Contact angles of various membranes (**a**) polyMPDSAH-g-PVDF membrane; (**b**) polyMEDSA-g-PVDF membrane.

The surface hydrophilicity, as one of the important properties, can affect the flux and anti-fouling ability of membranes [15,19]. In this work, the contact angle values of the modified membranes were measured to confirm the formation of polyMPDSAH and polyMEDSA on the PVDF membrane and to assess the hydrophilization effect of both grafted sulfobetaine polymers. Figure 5 shows the static contact angle for various membranes. On the one hand, due to the intrinsic hydrophobicity of PVDF, the contact angle of the nascent PVDF membrane is as high as 92.4°. After grafting with sulfobetaine polymers, the contact angle values decrease obviously due to the successful grafting of zwitterionic sulfobetaine polymers onto the chemically inert PVDF membrane surface. On the other hand, the contact angles drop below 25° and 60° for MPH-6-2-7 and MEA-6-2-7 with grafting amounts of 673.2 μg/cm^2^ and 225.7 μg/cm^2^, respectively. Furthermore, the results indicate that the hydrophilicity of the polyMPDSAH-g-PVDF membrane was superior to that of the polyMEDSA-g-PVDF membrane. This was attributed to the additional hydrophilic amide group (O=C–NH) in the molecular structure of MPDSAH and the larger grafting amount of sulfobetaine polymer on the outside surface of the polyMPDSAH-g-PVDF membrane.

### 2.4. Protein Adsorption

Protein adsorption was evaluated by incubating the membranes in a protein solution and determining the amount of adsorbed protein by measuring the reduced protein concentration via the Bio-Rad Protein Assay. The protein bovine serum albumin (BSA) was used in this work. Table 1 shows the protein adsorption on various membranes. BSA adsorption of the nascent PVDF membrane was 21.4 μg/cm^2^. In contrast, a hydration layer was formed on the polyMPDSAH-grafted or polyMEDSA-grafted membranes, which was considered to enhance surface resistance and reduce protein fouling. Due to the higher hydrophilicity of the polyMPDSAH-g-PVDF membrane compared with the polyMEDSA-g-PVDF membrane, protein adsorption of the former was lower, even for the same grafting amount condition (*i.e.*, MPH-3-2-7 and MEA-6-2-10).

### 2.5. Anti-Protein-Fouling Performance

#### 2.5.1. Cyclic Filtration of BSA Solution

**Figure 6:**
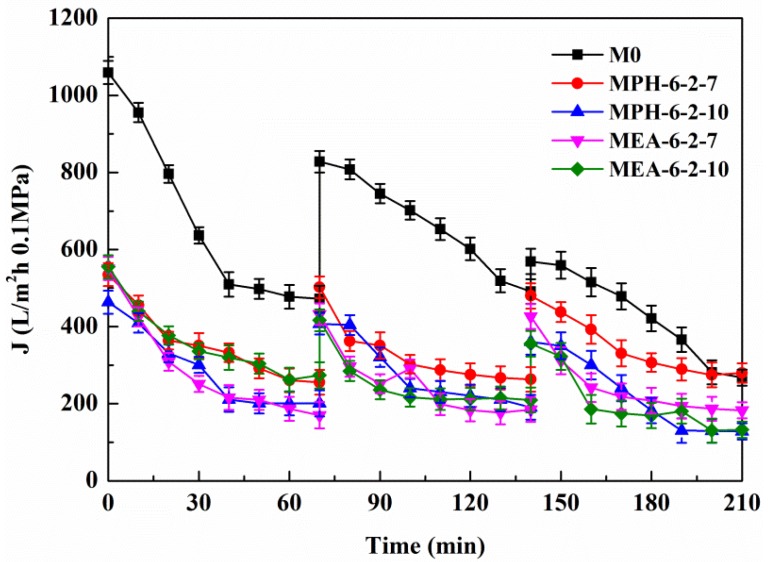
Permeate flux decline behavior of various membranes during cyclic filtration of a BSA solution.

Figure 6 shows the permeation fluxes of BSA solution through the various membranes. It can be seen that, for each cycle, the permeate fluxes of nascent and modified PVDF membranes decline rapidly at the start of the filtration. The relatively steady permeation fluxes are finally observed at a later stage in each filtration cycle, suggesting that the adsorption/deposition and the back diffusion of the protein molecules reached an equilibration at the membrane surface. The modified PVDF membranes with MPDSAH (MPH-6-2-7 and MPH-6-2-10) and MEDSA (MEA-6-2-7 and MEA-6-2-10) exhibit a much slower flux decline with time and the final steady permeate fluxes are much higher than the nascent PVDF membrane (M0). The results indicate that grafting of polyMPDSAH or polyMEDSA chains efficiently reduces the degree of flux decline caused by protein fouling on the PVDF membrane. Besides, the permeate flux decline of polyMPDSAH-g-PVDF membranes is smaller than the permeate flux decline of polyMEDSA-g-PVDF membrane due to the higher hydrophilicity.

In each cycle, after the BSA solution filtration, PVDF membranes were washed and then pure water fluxes were measured again. The values were used to calculate the relative flux recovery (*RFR_i_*_(*i* = 1,2)_), reversible fouling ratio (*RF_i_*), and irreversible fouling ratio (*IRF_i_*) [20,22]. The *RFR_i_*, defined as the ratio of pure water flux in the *i*th cycle to that in the initial process, can reveal the extent of cleaning efficiency and the effect of irreversible fouling resistance of the membrane [3,18]. The higher the value of *RFR_i_*, the lower the persistent protein adsorption onto the membrane during the *i*th cycle. Additionally, *RF_i_* can be recovered by hydraulic cleaning, but *IRF_i_* cannot. It is observed in Table 3 that the value of *RFR*_2_ is only 53.7% for M0 after two filtration cycles. But high values are achieved (more than 90% and 75%, respectively) for MPH-6-2-7 and MEA-6-2-7. This suggests that significant irreversible protein fouling occurred when the nascent PVDF membrane was used, while the poly(sulfobetaine)-g-PVDF membranes prevent the fouling phenomena. In this study, the highly hydrophilic zwitterionic groups of polyMPDSAH and polyMEDSA can take up high portions of free water on the membrane surface and form a water layer via electrostatic interaction, which provides a steric repulsion as the protein approaches the PVDF membrane surface. Besides, it is obvious that the capability of flux recovery for the polyMPDSAH-g-PVDF membrane is stronger compared with the polyMEDSA-g-PVDF membrane. In aqueous medium, the grafting chains with zwitterionic structure cannot diffuse into the interior of the protein molecules, while they can minimize the effect on exterior surface ions of proteins, and can maintain the “normal conformation” of protein molecules [25,26,27]. Therefore, the protein molecules have less chance to make contact with the poly(sulfobetaine)-g-PVDF membrane with a larger zwitterionic structure. To further analyze membrane fouling, the value of *RF_i_*/*IRF_i_*_(*i* = 1,2)_ was calculated to demonstrate the ratio of reversible fouling to irreversible fouling in each cycle. The data in Table 3 illustrate that the *RF_i_*/*IRF_i_*_(*i* = 1,2)_ of MPH-6-2-7 and MEA-6-2-7 are nearly 5 times and twice higher than that of M0, respectively. It indicates that part of irreversible fouling converts into the reversible fouling with the grafting of poly(sulfobetaine). Moreover, compared to polyMEDSA-g-PVDF membranes, polyMPDSAH-g-PVDF membranes can recover a higher water flux after hydraulic cleaning and show a lower membrane fouling during cyclic BSA filtration.

**Table 3:** The fouling-resistance of various membranes.

Membrane	*RFR*_1_ (%)	*RF*_1_/*IRF*_1_ (%)	*RFR*_2_ (%)	*RF*_2_/*IRF*_2_ (%)	*R* (%)
M0	78.2	1.2	53.7	0.6	18.5
M0 *	71.7	1.1	44.2	1.0	20.3
MPH-6-2-7	94.2	5.5	90.3	5.6	60.2
MPH-6-2-7 *	98.4	23.9	96.1	16.3	70.8
MPH-6-2-7 **	91.5	4.7	88.6	4.6	59.8
MEA-6-2-7	78.4	2.1	77.3	4	49.5
MEA-6-2-7 *	79.4	2.5	64	4.2	51.3
MEA-6-2-7 **	78	1.8	57.2	1.2	49.5

* Anti-fouling properties during the cyclic filtration of BSA solution with 100 mmol/L of NaCl; ** Anti-fouling properties during the cyclic filtration of BSA solution after cleaning in NaClO solution.

The rejection (*R*) is an important factor to assess the separation performance of membranes. The rejection of M0 is as low as 18.5%, while the values of MPH-6-2-7 and MEA-6-2-7 increase by more than three and two times, respectively. The higher grafting amount of polyMPDSAH from the PVDF hollow fiber membrane contributes to a much better retention performance of the polyMPDSAH-g-PVDF membrane.

#### 2.5.2. Cyclic Filtration of BSA Solution with NaCl

**Figure 7:**
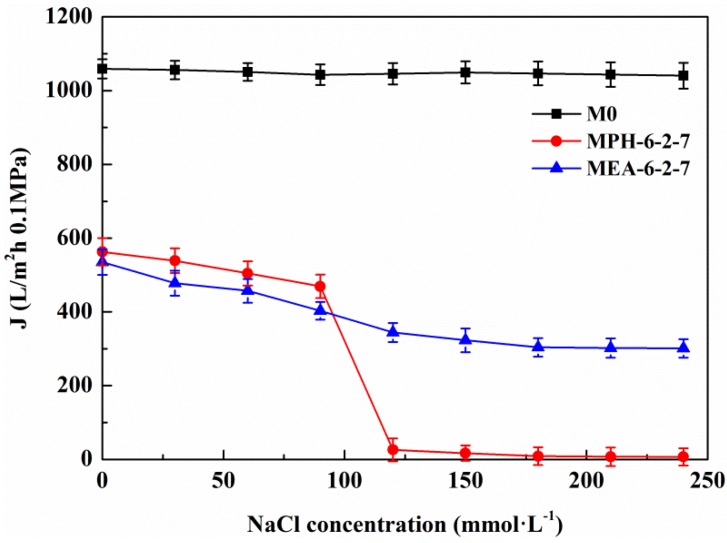
Permeate flux of aqueous solution with different NaCl concentrations through various membranes.

Based on the electrolyte-responsive behavior of the sulfobetaine polymer, NaCl in protein solution will induce swelling of poly(sulfobetaine) [23,28]. As a result, the surface morphology of the modified PVDF membrane transforms, which has a significant influence on the membrane permeability and fouling-resistance performance. Figure 7 demonstrates the permeate flux of the aqueous solution with different NaCl concentrations through various PVDF membranes. For M0, the permeate flux exhibits a behavior independent of the electrolyte concentration. The permeate flux of MEA-6-2-7 decreases gradually with increasing NaCl concentration, indicating a slightly electrolyte-responsive behavior. Conversely, the permeate flux of MPH-6-2-7 shows a sharp decrease at NaCl concentrations of 90 to 120 mmol/L. This means that a 90–120 mmol/L NaCl solution induces a full stretching of grafted MPDSAH polymer chains on the outside surface of MPH-6-2-7. Besides, the values of flux fell to 0 L/m^2^·h at NaCl concentrations of 120 mmol/L or above. Due to the high grafting amount of polyMPDSAH-g-PVDF membrane, the capability of the grafted polyMPDSAH to stretch or contract with the various of Na^+^ and Cl^−^ ions in the solution was relatively high. Therefore, the majority of pores had been blocked for liquid flow by the swelling of grafted polyMPDSAH when the NaCl concentration reached 120 mmol/L; that is, the MPH-6-2-7 shows an obvious electrolyte-responsiveness.

**Figure 8:**
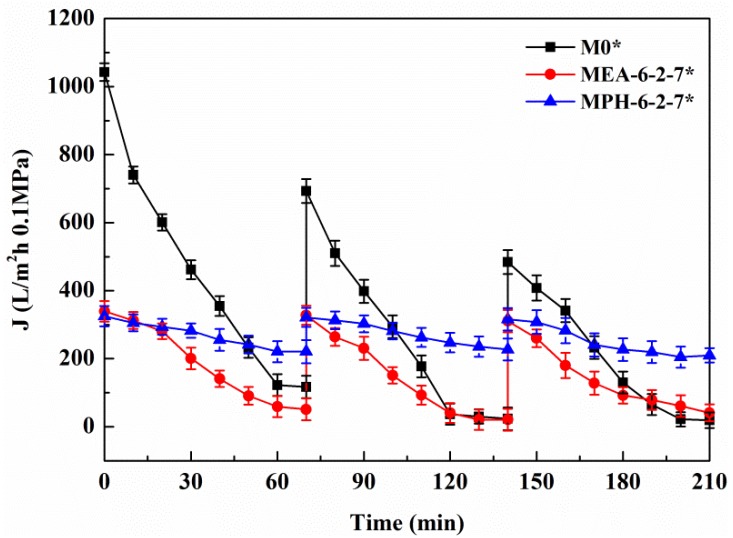
Permeate flux decline behavior of various membranes during the cyclic filtration of BSA solution with 100 mmol/L NaCl.

The permeate flux decline behavior of M0, MPH-6-2-7, and MEA-6-2-7 were examined in cyclic filtration of the BSA solution with a NaCl concentration of 100 mmol/L, as illustrated in Figure 8 and Table 3. For M0, the degree of permeate flux decline increases during cyclic filtration of the BSA solution with NaCl and the *RFR_i_*_(*i* = 1,2)_ and *RF_i_*/*IRF_i_*_(*i* = 1,2)_ are less than 75% and 1.5%, respectively. The stability of the protein solution is improved by small amounts of electrolyte, causing a higher membrane fouling for the nascent PVDF hollow fiber membrane. The value of permeate flux through MPH-6-2-7 decreases very slowly in the BSA solution containing 100 mmol/L of NaCl. The membrane (MPH-6-2-7*) shows a super-low fouling with *RFR_i_*_(*i* = 1,2)_ and *RF_i_*/*IRF_i_*_(*i* = 1,2)_ of more than 95% and 20%, respectively. However, the fouling resistance of MEA-6-2-7 is hardly improved during the filtration of the BSA solution with NaCl, because of the unobvious electrolyte-responsive behavior. Moreover, the *R* of MPH-6-2-7* for the protein increases further with the addition of NaCl, as shown in Table 3.

### 2.6. Stability

**Figure 9:**
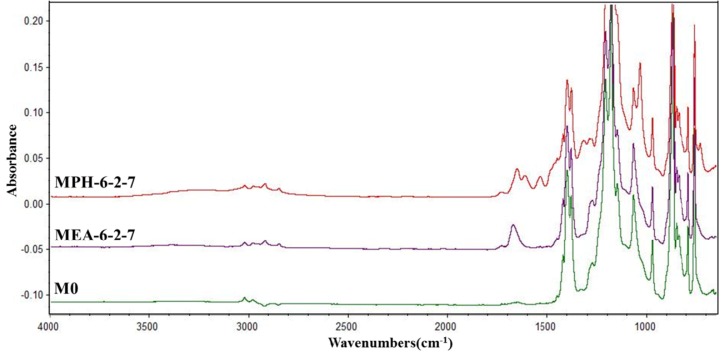
ATR-FTIR spectra of MPH-6-2-7 and MEA-6-2-7 after cleaned in 500 mg/L NaClO solution for 16 days.

The stability of the grafted polymer layer (polyMPDSAH or polyMEDSA) on the PVDF membrane was evaluated in sodium hypochlorite (NaClO) solution, which is usually used for membrane cleaning [28,29]. Figure 9 presents the ATR-FTIR absorbance spectra of the polyMPDSAH-g-PVDF and polyMEDSA-g-PVDF membranes after cleaned in NaClO solution. After cleaned for 16 days, the characteristic peaks of O–C=O and –SO_3_ in MEA-6-2-7 disappear, indicating the variation of the surface composition of polyMEDSA-g-PVDF membrane. But the ATR-FTIR absorbance for the introduced groups on the surface of MPH-6-2-7, such as –CH_2_–, –NH– and –SO_3_, still can be observed for cleaning, meaning a good stability of the grafted polyMPDSAH layer on the PVDF membrane surface.

**Figure 10:**
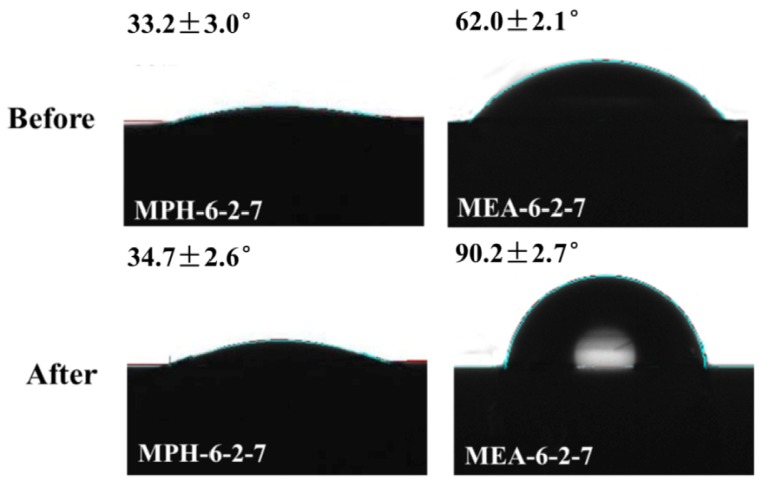
Contact angle images of MPH-6-2-7 and MEA-6-2-7 before/after cleaning in 500 mg/L NaClO solution for 16 days.

To support this result, contact angle values of the cleaned polyMPDSAH-g-PVDF and polyMEDSA-g-PVDF membranes were measured. Figure 10 displays the images of static water drops on both modified PVDF membranes. The contact angle values of MPH-6-2-7 do not change after cleaning and have a value of about 33°. The results prove that the grafted hydrophilic polyMPDSAH layer on the PVDF membrane surface is stable after membrane cleaning. However, the part of the water drop that faces the air enlarges and the contact angle of MEA-6-2-7 increases to a comparable value with the nascent PVDF membrane (CA of 90.4°, as shown in Figure 5), which indicates that the membrane loses the grafted hydrophilic polymer. The anti-protein-fouling performance of the membranes after cleaning in sodium hypochlorite solution was studied, as illustrated in Table 3. *RFR_i_*_(*i* = 1,2)_ and *RF_i_*/*IRF_i_*_(*i* = 1,2)_ of MPH-6-2-7** remain practically unchanged after 16 days membrane cleaning. For MEA-6-2-7**, increasing the cleaning time decreases *RFR_i_*_(*i* = 1,2)_ and *RF_i_*/*IRF_i_*_(*i* = 1,2)_, which demonstrates a decline of anti-protein-fouling property.

This study clearly demonstrates that the loss or hydrolysis of the grafted MPDSAH polymer layer on the polyMPDSAH-g-PVDF membrane is negligible. Compared with the polyMEDSA-g-PVDF membrane, the polyMPDSAH-g-PVDF membrane exhibits a much better stability. It can be ascribed to the amide bond in MPDSAH, which is more stable than the ester bond in MEDSA [18,30]. It can be concluded that the additional amide group of sulfobetaine plays an important role for stable anti-protein-fouling performance in the modification of the PVDF hollow fiber membrane via the two-step polymerization.

## 3. Experimental Section

### 3.1. Materials

Poly(vinylidene fluoride) (PVDF) hollow fiber membranes were prepared by the method described in our preceding study [1]. The used chemicals, 2-(methacryloyloxyethyl)ethyl-dimethyl-(3-sulfopropyl)-ammonium (MEDSA), 3-(methacryloylamino)propyl-dimethyl-(3-sulfopropyl) ammonium hydroxide (MPDSAH) and *N*,*N*′-methylene bisacrylamide (MBAA) were obtained from Beijing Hengye Zhongyuan Chemical Co., Ltd. (Beijing, China). Sodium chloride (NaCl) was purchased from Sinopharm Chemical Reagent Beijing Co., Ltd. (Beijing, China). All reagents were used as received.

### 3.2. Preparation of PolyMPDSAH-g-PVDF and PolyMEDSA-g-PVDF Membrane

#### 3.2.1. ATRP Initiated Directly from PVDF Membrane Outside Surface

The PVDF hollow fiber membrane was rinsed successively with ethanol and deionized water at 40 °C. After both ends were tied up, the PVDF membrane was dried under reduced pressure at room temperature for 24 h prior to the surface modification reaction. The PVDF membrane was soaked in methanol for 1 min to wet out and placed in a dry flask. Then, HEMA (8 g, 61.5 mmol), CuCl_2_ (30.8 mg, 0.22 mmol), PMDETA (46 µL, 0.22 mmol) and pure water (43 mL) were added sequentially to the flask under nitrogen protection at 40 °C. After 30 min, a degassed solution of AscA (0.03 mmol/mL, 2.4 mL, 66 mol) was transferred into the flask under nitrogen protection and polymerization was allowed to continue for 6 h. The modified PVDF hollow fiber membrane (polyHEMA-g-PVDF) was removed and washed with ethanol at 40 °C for 24 h, followed by washing with water at 40 °C for another 24 h. The membrane (polyHEMA-g-PVDF) was then dried under vacuum at 40 °C for 48 h.

#### 3.3.2. Ce (IV)-Induced Graft Copolymerization on the PVDF Membrane

The dried polyHEMA-g-PVDF membranes were placed in a dry flask and then mixed with 50 mL degassed solution containing CAN (0.04~0.10 mol/L), HNO_3_ (0.4~1.0 mol/L), MPDSAH (0.2~0.4 mol/L), and MBAA (0~0.06 mol/L). Subsequently, the system was heated at 40 °C for 3 h. The grafted membranes were thoroughly washed with phosphate-buffered saline (PBS, pH = 7.4) and a mixture of NaH_2_PO_4_·2H_2_O and Na_2_HPO_4_·12H_2_O. After rinsing with water, the membranes were dried under vacuum at 40 °C for 24 h and finally weighted.

The grafting amount (GA, μg/cm^2^) was calculated as follows:

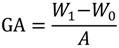
(1)
where *A* represents the efficient surface area of the PVDF membrane (cm^2^), *W*_0_ and *W*_1_ are the weights of the nascent and modified PVDF membrane (μg), respectively. Each result was an average of at least three independent experiments. The difference between both sulfobetaine monomers and the resulting membrane are listed in Table 2 and Table 4, respectively.

**Table 4:** Preparation conditions of various membranes.

Membrane code	Membrane	MBAA (mol/L)	Sulfobetaine concentration		CAN (mol/L)
			MPDSAH (mol/L)	MEDSA (mol/L)	
M0	PVDF	–	–	–	–
MPH-0-2-7	polyMPDSAH-g-PVDF	0	0.2	–	0.07
MPH-3-2-7	polyMPDSAH-g-PVDF	0.03	0.2	–	0.07
MPH-6-1-7	polyMPDSAH-g-PVDF	0.06	0.1	–	0.07
MPH-6-2-7	polyMPDSAH-g-PVDF	0.06	0.2	–	0.07
MPH-6-3-7	polyMPDSAH-g-PVDF	0.06	0.3	–	0.07
MPH-6-2-4	polyMPDSAH-g-PVDF	0.06	0.2	–	0.04
MPH-6-2-10	polyMPDSAH-g-PVDF	0.06	0.2	–	0.10
MEA-0-2-7	polyMEDSA-g-PVDF	0	–	0.2	0.07
MEA-3-2-7	polyMEDSA-g-PVDF	0.03	–	0.2	0.07
MEA-6-1-7	polyMEDSA-g-PVDF	0.06	–	0.1	0.07
MEA-6-2-7	polyMEDSA-g-PVDF	0.06	–	0.2	0.07
MEA-6-3-7	polyMEDSA-g-PVDF	0.06	–	0.3	0.07
MEA-6-2-4	polyMEDSA-g-PVDF	0.06	–	0.2	0.04
MEA-6-2-10	polyMEDSA-g-PVDF	0.06	–	0.2	0.10

### 3.3. Membrane Characterization

The surface chemical composition of the nascent and modified PVDF membranes were characterized by using attenuated total reflectance spectrophotometry on a Nicolet 6700 ATR-FTIR (ThermoFisher Scientific, Waltham, MA, USA) and X-ray photoelectron spectroscopy on an ULVAC-PHI XPS (ULVAC-PHI, Kanagawa, Japan) [20,22,23].

The surface morphologies of the nascent and modified PVDF membranes were observed using a JEOL JSM 7401scanning electron microscope (JEOL, Tokyo, Japan) under standard high-vacuum conditions (1 kV). All the samples were frozen in liquid nitrogen, then deposited onto a copper holder and sputtered with gold prior to SEM. The pore size of the outside surface of the hollow fiber membranes was analyzed by using the software package Image-Pro Plus 6.0 (Media Cybernetics, Silver Spring, MD, USA) and SmileView (JEOL, Tokyo, Japan).

Tensile strength (MPa) and elongation ratio (%) of the nascent and modified PVDF hollow fiber membranes were measured on a Shimadzu AGS-J 20N (Shimadzu, Kyoto, Japan) at a loading velocity of 3 cm/min. The reported data are the average values of three experimental runs.

The thermal properties of the hollow fiber membranes were determined by Differential Scanning Calorimetry (DSC) on a TA Q200 (TA Instruments, New Castle, PA, USA). The DSC sample was heated from 50 °C to 200 °C with a heating rate of 10 °C/min.

Static contact angle between water and PVDF hollow fiber membranes was measured and calculated on an OCA 20 contact angle goniometer (DataPhysics Instruments GmbH, Filderstadt, Germany) at 25 °C. Water (1 µL) was carefully dropped onto the dry sample with an automatic piston syringe for 20 s and then the contact angle was measured. The contact angle value of each sample was measured at three different positions on each sample.

A Bio-Rad Protein Assay (Bio-Rad Laboratories, Hercules, CA, USA) was used to evaluate the protein adsorption of bovine serum albumin (BSA) on the nascent and modified PVDF membranes. Membranes with 20.41 cm^2^ of efficient surface area were rinsed with ethanol (20 mL) for 30 min and transferred to a test tube with PBS solution (20 mL, pH = 7.4). After 30 min, the membrane was soaked in protein solutions (1.0 g/L, 5 mL), which were prepared in PBS, for 24 h at 37 °C. Then, dye reagent-containing coomassie brilliant blue G-250 was added to the solutions. A Spectrumlab 22PC visible-infrared spectrometer (Shanghai Arris light technology Ltd., Shanghai, China) was used to determine the solution concentration. The protein adsorption (μg/cm^2^) of the membranes was calculated according to the following equation:

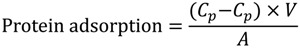
(2)
where *C*_0_ and *C*_1_ are the protein (BSA) concentration in PBS solution before and after soaking the membrane (μg/mL), *V* is the volume of the soaking solution (mL), and *A* is the effective area of the PVDF membrane (cm^2^).

### 3.4. Anti-Fouling Performance

A self-made dead end filtration was applied to characterize the cyclic filtration performance of PVDF hollow fiber membranes. BSA solution (0.1 g/L) was used as a model protein solution to evaluate the protein resistance characteristics of the membranes under a pressure of 0.1 MPa. Details of the general procedure have been described previously [20,22,23]. Additionally, the flux of NaCl aqueous solution (*J*_NaCl,0_) was measured at NaCl concentrations of 0–240 mmol/L. Cyclic filtration was performed at a NaCl concentration in BSA solution of 100 mmol/L. To evaluate the fouling-resistance of the membrane, the rejection (*R*, %), relative flux recovery (*RFR_i_*, %), the reversible (*RF_i_*, %) and irreversible protein fouling (*IRF_i_*, %) in each cycle of the filtration experiments were calculated according to the following equations [20,22,23]:

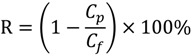
(3)

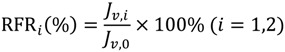
(4)

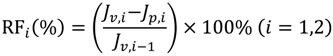
(5)

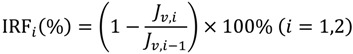
(6)


### 3.5. Stability Test

The stability of the grafted sulfobetaine layer was evaluated by monitoring the surface composition and anti-protein-fouling performance of the modified PVDF membranes after membrane cleaning in 0.5 g/L sodium hypochlorite (NaClO) solution. After 16 days, each sample was taken out and rinsed with pure water. Then, ATR-FTIR supported by CA measurements and the filtration test were carriet out on the dried sample to identify changes in the surface composition and anti-fouling performance.

## 4. Conclusions

In this study, a highly hydrophilic and anti-protein-fouling PVDF hollow fiber membrane with a good stability was prepared by a two-step polymerization with sulfobetaine monomers. The ATR-FTIR, XPS, and SEM analyses confirmed that the two sulfobetaine polymers (polyMPDSAH and polyMEDSA) were successfully grafted onto the PVDF membrane. Based on the larger molecular weight, the higher melting point, and the additional amide group, the polyMPDSAH-g-PVDF membrane showed a higher grafting amount, better thermal properties, and hydrophilicity than the polyMEDSA-g-PVDF membrane. When the grafting amount of polyMPDSAH and polyMEDSA reached 673.2 μg/cm^2^ and 225.7 μg/cm^2^, respectively, the contact angle of both modified PVDF membranes became constant. It may be concluded that the concentration of the cross-linking agent, zwitterionic monomer, and initiator at 0.6, 0.2 and 0.07 mol/L, respectively, were the optimum conditions for producing a product with improved membrane strength and hydrophilicity. By conducting cyclic filtration experiments with a BSA solution, the modified PVDF membranes were shown to possess improved anti-protein-fouling properties compared with the nascent PVDF membrane. Due to the high hydrophilicity and the obvious electrolyte-responsive behavior, the polyMPDSAH-g-PVDF membrane showed a super-low fouling with *RFR_i_*_(*i* = 1,2)_ and *RF_i_*/*IRF_i_*_(*i* = 1,2)_ of more than 95% and 15%, respectively. After cleaned for 16 days in NaClO solution, the polyMEDSA-g-PVDF membrane lost its grafted polymers and showed a declined anti-protein-fouling performance; however, almost no changes in the surface composition and anti-protein-fouling performance were found for the polyMPDSAH-g-PVDF membrane due to the good chemical stability of the amide groups (O=C–NH) in this membrane. Therefore, the additional amide group of sulfobetaine played an important role in the modification of the PVDF hollow fiber membrane via the two-step polymerization, which resulted in a novel membrane with excellent membrane strength, good thermal properties and anti-protein-fouling performance with long-term stability.
